# High-Resolution Melting (HRM) Analysis of the Cu/Zn Superoxide Dismutase (SOD1) Gene in Japanese Sporadic Amyotrophic Lateral Sclerosis (SALS) Patients

**DOI:** 10.1155/2011/165415

**Published:** 2011-04-12

**Authors:** Chizuru Akimoto, Mitsuya Morita, Naoki Atsuta, Gen Sobue, Imaharu Nakano

**Affiliations:** ^1^Division of Neurology, Department of Internal Medicine, Jichi Medical University, 3311-1 Yakushiji, Shimotsuke-shi, Tochigi 329-0498, Japan; ^2^Department of Neurology, Nagoya University Graduate School of Medicine, 65 Tsurumai-cho, Showa-ku, Nagoya-shi, Aichi 466-8550, Japan

## Abstract

Amyotrophic lateral sclerosis (ALS) is a progressive neurodegenerative disorder, and the majority of ALS are sporadic (SALS). Recently, several causative genes for familial ALS (FALS) were identified, but the cause of the SALS is still unknown. This time, we aimed to identify the genetic background of SALS. First, we applied the new sensitive screening methods: high-resolution melting (HRM) analysis. HRM analysis detected 18 out of 19 known SOD1 gene mutations (94.7% sensitivity). Next, we screened *SOD1*, three novel mutations (C6Y, Q22H, and S134T) were identified in our own 184 SALS cases (1.63% prevalence), and four mutations in another 255 SALS cases (1.56% prevalence) registered from all over Japan. The patients with *SOD1* mutations suggested a relatively young onset and limb involvement at onset. The HRM analysis is a sensitive and easy screening method; we will use this method for screening other ALS causative genes and revealing the genetic background of SALS.

## 1. Introduction

 Amyotrophic lateral sclerosis (ALS) is a neurodegenerative disorder primarily affecting motor neurons in the spinal cord, brain stem, and cerebral cortex. Five to ten percent of ALS cases are familial; the others are believed to be sporadic [1]. Mutations in the Cu/Zn superoxide dismutase gene (*SOD1*; OMIM 147450) are the most frequent genetic defects known to underlie ALS, accounting for 20% of familial cases (FALS) and one to seven percent of apparently sporadic cases (SALS) [1–7]. Recently, other mutations like the TARDBP gene (TDP-43) [8, 9], ANG gene [10], FUS/TLS gene [11], and OPTN gene [12] were identified as causative of ALS. Despite this genetic heterogeneity, *SOD1* mutations are the most frequent cause of adult onset ALS. Here, we report the results of screening for *SOD1* mutations in the 184 SALS cases in our hospital and 265 ALS cases all over Japan by high-resolution melting (HRM) analysis.

 HRM analysis is a mutation scanning technique that monitors the progressive change in fluorescence caused by the release of an intercalating DNA dye from a DNA duplex as it is denatured with marginal increases in temperature [13]. The shifts and shapes of melting curves, there are obtained as fluorescence difference plots, are used to distinguish between mutations and controls. HRM analysis of PCR products amplified in the presence of LC Green Plus can detect all heterozygous and most homozygous sequence variations through differences in shape and position of a melting curve compared with a wild-type melting profile. Although single-strand conformation polymorphism (SSCP) [2, 3, 14–20] and denaturing high-performance liquid chromatography (DHPLC) [5, 6] seem to be the main screening strategies for *SOD1* mutations, HRM analysis has its own advantages. This is the first report of HRM analysis being applied to the *SOD1* screening. In this paper, we report the high sensitivity of HRM analysis for known *SOD1* mutations, and the prevalence and clinical features of *SOD1* mutations in Japanese SALS cases.

## 2. Patients and Methods

### 2.1. Patient Group 1

 A total of consecutive 184 SALS cases (109 males and 75 females) visited our Neurology Division at the Jichi Medical University Hospital in Tochigi, Japan. Ethical approval was granted by the Bioethics Committee for Human Gene Analysis of our university and informed consent was obtained from all subjects according to the Declaration of Helsinki. Every patient fulfilled the diagnostic criteria for ALS as outlined by the *El Escorial Revisited* [21] classification; 177 definite, probable or possible ALS and 7 suspected ALS. None of the cases had a family history of a neuromuscular disorder. There was no significant difference in onset age between 109 males and 75 females (males: 60.4 years on average with a range of 27–80; females: 64.3 years with a range of 34–83).

### 2.2. Patient Group 2

 In 2006, the Japanese Consortium for Amyotrophic Lateral Sclerosis Research (JaCALS) was organized with the aim of investigating the relationships of clinical and genetic aspects of ALS in Japan. The Ethics Committee of each institution granted ethical approval. The inclusion criteria for registration with the JaCALS are: (1) adult onset, steady progressive course, (2) definite, probable or possible ALS based on the *El Escorial Revisited* [21] criteria for diagnosis of ALS, and (3) informed consent for the genetic study and clinical checking every three months. From 2006 to 2008, 265 patients (10 FALS and 255 SALS) were registered, and blood samples and clinical data having been obtained by neurologists.

### 2.3. Reported SOD1 Mutations

We used 19 reported *SOD1* mutations in all five exons (Table 1) to determine the sensitivity of the HRM analysis. 19 reported *SOD1* mutations were obtained from our collaborators, Dr. Andersen P. (Umeå University, Sweden) and Dr. Watanabe Y. (Tottori University, Japan), and they were already direct sequenced and confirmed they had the mutations.

### 2.4. HRM Analysis and Sequencing

 Genomic DNA was extracted from lymphocytes using a standard procedure. We designed PCR primers for HRM analysis to screen all five exons in *SOD1*. DNA samples were amplified with double-stranded DNA-binding dye LC Green Plus (Idaho Technology). PCR was performed with a Veriti 96-Well Thermal Cycler (Applied Biosystems) in 10 *μ*L reaction mixtures comprising 10 ng DNA, 1XPCR buffer, LC Green Plus (Idaho Technology), and 1 U Taq polymerase, with 0.25 *μ*M each forward and reverse primers. Initial denaturation was performed at 95°C for 2 min, followed by 45 cycles of 94°C for 30 sec and 62–68°C for 30 sec, with a final cycle of 94°C for 30 sec and 25°C for 30 sec.

 We performed melting acquisition with a 96-well Light Scanner (Idaho Technology). The plate was heated from 80 to 98°C at 0.1°C/sec with a 300 ms frame interval, 15 ms exposure, and 100% LED power. Light Scanner Software was used for melting curve analysis. The Light Scanner analyses of 96 samples were performed in around 10 min. Sequencing of samples indicated to include mutations was then carried out using a BigDye Terminator v1.1 Cycle Sequencing Kit (Applied Biosystems) and an ABI 310 automated sequencer (PE Applied Biosystems).

 First we examined 19 reported *SOD1* mutations to determine the sensitivity of HRM analysis. Next, we applied this method to Japanese ALS patients for mutation screening of *SOD1*.

## 3. Results

### 3.1. Sensitivity of HRM Analysis

**Figure 1:**
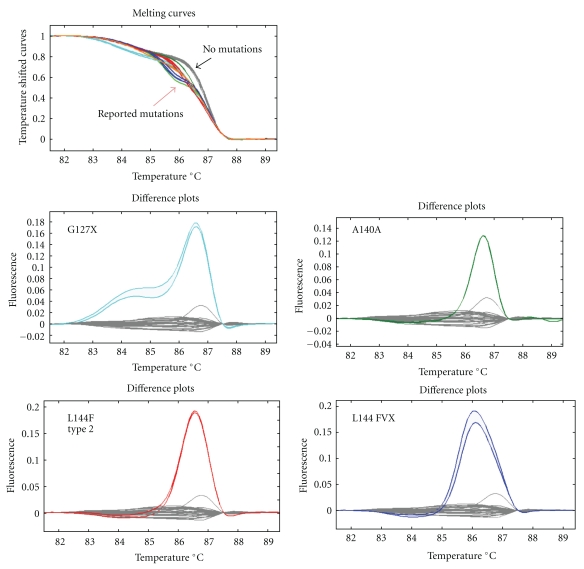
Melting curves and subtractive fluorescent difference plots of a wild type (gray lines) and reported *SOD1* mutations (colour lines). Difference plots were easily identified for the mutations.

 HRM analysis clearly distinguished 18 of 19 previously identified *SOD1* mutations from normal controls. The mutation detection sensitivity was 94.7% for the reported mutations. The melting curves of control samples (wild-type) were tightly grouped for all fragments, and altered difference curves were easily identified for the 18 mutations (Figure 1). The mutation that could not be detected was Gly 114 Ala.

### 3.2. SOD1 Mutations and the Clinical Characteristics in Group 1

**Figure 2:**
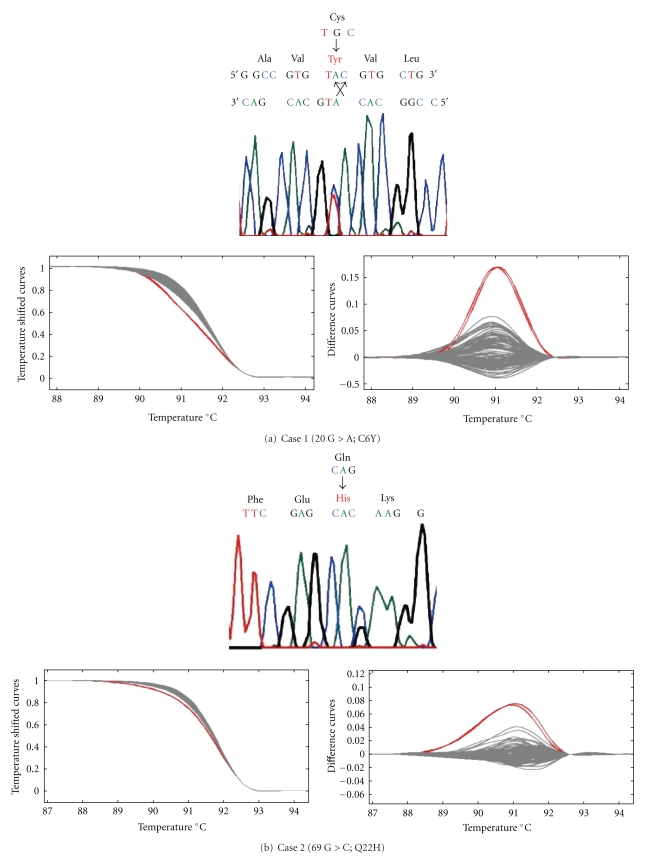

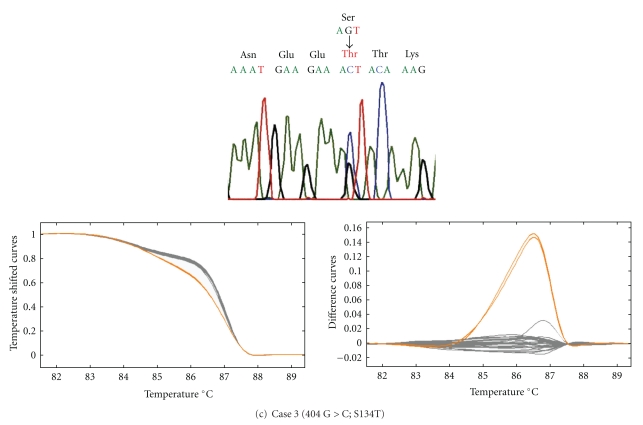
Sequence (upper), melting curves (left lower) and subtractive fluorescent difference plots (right lower) of the three novel mutations.

We found *SOD1* mutations in three out of the 184 SALS cases (1.63%) in the group 1. The mutations identified were all novel: Cys 6 Tyr (C6Y) and Gln 22 His (Q22H) in exon 1, and Ser 134 Thr (S134T) in exon 5 (Figure 2).

 In case 1, a 34-year-old woman, there was a single-base pair substitution in exon 1 at codon 6 (TGC to TAC). This change created a cysteine 6 to tyrosine missense mutation (C6Y). She awoke with painful cramping and weakness in the right leg almost every morning at the age of 33 years. The cramping resolved, but her right leg weakness progressed and become accompanied by fasciculation. One year after the onset, neurological examination showed marked muscle atrophy and prominent fasciculation in her right leg. Tendon reflexes were normal, and plantar responses were flexor. Sensations in all four modalities were intact. Nerve conduction studies revealed mild reduction of motor nerve conduction velocity without conduction block. Needle electromyographic analysis showed repetitive discharges and hyperexcitability only in the right leg. Extensive screening for causes of the motor neuropathy was negative. The muscle weakness and atrophy progressed, and spread to the other parts of her body despite treatment with intravenous gamma globulin, cyclophosphamide, and plasmapheresis. The disease course was rapid and the bulbar symptom developed in the last stage. She expired 3 years after disease onset.

 In case 2, a 48-year-old man, there was a single-base pair substitution in exon 1 at codon 22 (CAG to CAC). This change created a glutamine 22 to histidine missense mutation (Q22H). He developed left leg weakness and atrophy at the age of 46 years. Two years after the onset, neurological examination showed muscle weakness, atrophy and fasciculation were observed in the left leg. Tendon reflexes were brisk in the right leg and both arms. The weakness and atrophy spread to the right leg, confining him to a wheelchair at 51 years old and to bed at 52 years old. He underwent tracheotomy because of progressive respiratory failure, and artificial ventilation support was started eight years after disease onset. Five years after artificial ventilation support was started, he moved to another hospital and thus we could not follow him further.

 In case 3, a 69-year-old man, there was a single-base pair substitution in exon 5 at codon 134 (AGT to ACT). This change created a serine 134 to threonine missense mutation (S134T). He noticed gait disturbance due to muscle weakness of the lower limbs at the age of 62 years. The weakness progressively worsened, and he could not walk by himself at 67 years old. Neurological examination showed muscle weakness, and fasciculation were evident in the upper and lower limbs. Tendon reflexes were diminished and plantar responses were flexor. No sensory abnormalities were noted. Nerve conduction studies demonstrated normal motor and sensory nerve conduction velocities. Electromyographic analysis revealed fasciculation and denervation in the upper and lower limbs. Although upper motor neuron impairment was not confirmed, ALS was considered as the most probable diagnosis. The weakness progressed very slowly, and he died of respiratory insufficiency seven years after disease onset.

### 3.3. SOD1 Mutations in Group 2

 We found *SOD1* mutations in eight out of 265 cases. Of these, four had family histories, mutations being Leu 38 Val (L38V) and His 46 Arg (H46R) in exon 2, Gly 93 Ser (G93S) in exon 3 and Gly 141 Ala (G141A) in exon 5. The G141A found in a woman whose brother probably died of ALS was a novel mutation. In this case, left hand weakness occurred at 57 years old. The clinical course was rapid that she died at 3 years and 11 months after the onset. The remaining four *SOD1* mutations were found in sporadic cases, mutations being Lys 3 Glu (K3E) in exon 1 and Gly 93 Ser (G93S) in exon 3. K3E was a novel mutation found in a woman who noticed right leg weakness at 52 years old, and artificial ventilation support was started 6 years after the onset. The G93S mutation was found in three unrelated patients. The prevalence of *SOD1* mutations in the SALS cases was 1.56% (4 of 255 SALS cases) in the group 2.

## 4. Discussion

### 4.1. HRM Analysis on SOD1

This is the first report of HRM analysis for *SOD1* mutation screening. HRM analysis could clearly distinguish 18 of 19 reported *SOD1* mutations from normal controls. We have demonstrated that HRM analysis is a rapid and sensitive (94.7% sensitivity) method for mutation scanning of *SOD1*. SSCP is a method that most laboratories use for the screening of gene mutations, but the sensitivity is not high (80% to 90%) [7]. DHPLC using WAVE system is also a screening method, but it cannot detect the D90A mutation [6], which is one of the worldwide detected *SOD1* mutations, and the most appropriate condition for analysis is difficult to determine. Using HRM analysis, we can analyze within 5 to 10 minutes on 96 samples and the running cost is not expensive.

 The one mutation that HRM analysis could not detect was guanine to cytosine at nucleotide 341 substituting glycine (GGC) to alanine (GCC) at codon 114. On the other hand, guanine (TTG) to cytosine (TTC) mutations (L144F), and alanine (GCT) to alanine (GCA) mutations (A140A) in other samples were detected with this method, indicating the possibility that the G to C mutation detection failure may be a sequence-specific phenomenon.

### 4.2. SOD1 Mutations in SALS

 We applied this method to our own 184 (group 1) and 255 (group 2) Japanese cases of SALS, finding three different novel *SOD1* mutations in three cases in the former (mutation prevalence, 1.63%), and one novel and three known mutations in four cases in the latter (mutation prevalence, 1.57%). We listed the prevalence and identified mutations of *SOD1* in SALS cases in other countries (Table 2). The prevalence was high in the Scottish population (7%) and widely ranged in Italy (0%–6%), but in other countries, it was 2 to 4%, similar to our data. This time we found four novel mutations in SALS cases, and these mutations were not found in the Japanese control group.

 In a sporadic ALS patient carrying an *SOD1* mutation, it is also difficult to ascertain whether it is a genuine sporadic case, a case due to a mutation, or a familial case with incomplete penetrance. To date, an SALS case with H80A is the only one with a proven de novo mutation [23]. In our analysis, the G93S mutation was found in three unrelated patients from the Tokai district of Japan (personal communication). There are at least 6 Japanese families with G93S, 4 of the 6 families being reported to be residents of the Tokai district [24–26]. The accumulation of G93S in Japanese SALS cases suggests the possibility of decreased penetrance or an incomplete family history rather than a de novo mutation.

### 4.3. Clinical Characteristics of SALS Involving SOD1 Mutations

Clinical characteristics such as onset age, onset symptoms, and clinical course of so far reported SALS patients having *SOD1* mutations are summarized in Table 3. Since A4V, D90A, and I113T have been observed worldwide and are considered to be the most common mutations in both familial and sporadic ALS cases [4, 7]. Because of the difficulty to define true sporadic, we did not include these three mutations in the table. Based on the results of analysis of these 20 *SOD1* mutations in 27 sporadic ALS patients (13 men, 10 women, and 4 unknown), the average age at onset was 43.8 (range 18–77) years, which is about 10 years younger that the mean age at onset reported for the sporadic ALS population [22]. The onset symptom was limb weakness in 21 cases and bulbar dysfunction only in one case. The clinical courses were under three years (rapid) in seven cases, over six years (slow) in nine cases, and three to six years (moderate) in five cases. The clinical characteristics of SALS involving *SOD1* mutations indicate a relatively young onset age and a high percentage of limb involvement at onset. These characteristics are similar to the features of ALSOD (ALS patients having *SOD1* mutations), not those of sporadic ALS [29].

 The C6Y mutation in our case was difficult to diagnose because the main symptom was lower motor neuron dysfunction and the onset age was young (midthirties). But this clinical course was similar to that in the case of de novo mutation H80A [23]. There were nine (bold) patients whose onset ages were under forty, and eight of them had rapid or moderate clinical course (Table 3). On the other hand, there are four (underlined) patients whose onset ages were over 55, and three of them had slow clinical course (Table 3). Gamez and his colleagues reported [4] there were three types of sporadic ALS patients who were particular candidates for genetic testing for *SOD1*: (a) those with the typical Scandinavian phenotype, (b) those with clinical onset before 55 years of age, and (c) patients with slow progression/long survival. Compare with this theory (b) and (c), only one patient (N19S) is an exception for *SOD1* screening. 

## 5. Conclusion

 We have demonstrated that HRM analysis is a rapid and sensitive method for the mutation scanning of *SOD1*. With this method, four novel *SOD1* mutations were found in SALS cases, the prevalence of *SOD1* mutations in Japanese SALS cases being 1.6%. The clinical characteristics of SALS involving *SOD1* mutations are a young onset age and a high percentage of limb involvement at onset. We will screen other causative genes for ALS (*TDP-43, ANG, FUS/TLS, OPTN* and others) by HRM analysis and determine the cause of disease appearance.

## Figures and Tables

**Table 1:** Reported *SOD1* mutations to determine the sensitivity of HRM analysis.

Exon1	A4V, L8V, V14G
Exon2	H43R
Exon3	D76Y
Exon4	N86S, A89V, D90A (hetero), G93S, D101G, S105L, G114A, R115G
Exon5	L126delTT, G127X, A140A, L144F type2, L144FVX

Underlined mutation could not detect the mutation by HRM analysis.

**Table 2:** *SOD1* mutations in SALS patients of the different countries.

Country	Total SALS	No. of* SOD1 *	*SOD1/*Total	Mutations identified	Screening method	Author, year
North England	46	1	2.1	D101N	SSCP	Jones et al. 1994 [14]
Scotland	57	4	7.0	E21K, I113T	SSCP	Jones et al. 1995 [2, 15]
Scandinavia	355	14	3.9	V14G, D90A (hetero & homo)	SSCP	Andersen et al. 1997 [16]
England	155	4	2.6	D90A, I113T, V118KTGPX	SSCP	Jackson et al. 1997 [17]
England	175	5	2.8	G72S	SSCP	Shaw et al. 1998 [18]
Belgium	69	3	4.3	D90A, N139N, IVS + 19A > G	SSCP	Aguirre et al. 1999 [3]
Italy	48	3	6.3	D90A (homo), I113T, A95T	DS	Gellera et al. 2001 [22]
Spain	87	1	1.2	N65S	SSCP	García-Redondo et al. 2002 [19]
Italy	225	0	0		SSCP	Batlistini et al. 2005 [20]
Spain (Catalonia)	94	4	4.2	D90A, N139H, A140A	DS	Gamez et al. 2006 [4]
Italy	66	3	4.5	K135X, N65S, A95T	DHPLC	Corrado et al. 2006 [5]
Italy	303	2	066	N19S, E133ΔE	DHPLC	Chiò et al. 2008 [6]
Japan	184	3	1.6	C6Y, Q22H, S134T	HRM	This article group1
Japan	255	4	1.5	K3E, G93S	HRM	This article group2

Total	2119	51	2.4			

DS: direct sequence (no screening method in the article).

**Table 3:** Clinical characteristics of the SALS patients having *SOD1* mutations.

Amino acid change	Sequence change	No. of pt.	Onset age	Onset symptom	Disease course/Disease duration	Author/Reference
K3E	AAG > GAG	1	52	Right leg weakness	Moderate, 6y	This article
C6Y	TGC > TAC	1	**34**	Right leg weakness	Moderate, 3y	This article
V14G	GTG > GGG	1	**39**	Both legs fatigue	ND, 16m~	Andersen et al. [16]
G16S	GGC > AGC	1	**18**	Hand paresis	Rapid, 1y	Kawamata et al. [27]
N19S	AAT > AGT	2	**32** 41	Both legs weakness Left arm weakness	Moderate, 36m ND	Mayeux et al. [28]
		1	77	Hand paresis	Rapid, 15m	Chiò et al. [6]
E21K	GAG > AAG	1	ND	ND	ND	Jones et al. [2]
Q22H	CAG > CAC	1	46	Left leg weakness	Slow, 8y	This article
N65S	AAT > AGT	1	44	Left leg weakness	Slow, 14y	García-Redondo et al. [19]
		1	40	Drop foot	Slow, 11y	Corrado et al. [5]
G72S	GGT > AGT	1	**29**	Left leg weakness	Rapid, 15m	Shaw et al. [18]
H80A	CAT > CGT	1	**24**	Left leg weakness	Rapid, 18m	Alexander et al. [23]
G93S	GGT > AGT	3	44 55 64	Both legs weakness Left leg weakness Right leg weakness	ND, 6y~ Slow, 8y~ Slow, 12y~	This article
A95T	GCC > ACC	1	**26**	Both legs weakness	Slow	Gellera et al. [22]
		1	45	Left drop foot	Slow, 20y	Corrado et al. [5]
D101N	GAT > AAT	1	53	ND	ND	Jones et al. [14]
V118 KTGPX	GTG > AAAACTG	1	**34**	ND	Rapid, 16m	Jackson et al. [17]
E133ΔE	GAA del GAA	1	54	Left leg weakness	Moderate, 4y	Chiò et al. [6]
S134T	AGT > ACT	1	62	Both legs weakness	Slow, 7y	This article
K136X	AAG > TAG	1	45	Left leg weakness	Rapid,12m	Corrado et al. [5]
N139H	AAG > CAC	1	53	ND	ND	Gamez et al. [4]
N139N	AAC > AAT	1	**33 **	ND	Moderate, 3y	Aguirre et al. [3]
A140A	GCT > GCA	2	52 ND	Bulbar palsy Limb weakness	Rapid, 22m Slow	Gamez et al. [4]

Total/Average	20	27	43.8	21 Extremity	7 Rapid
				1 Bulbar	5 Moderate
				5 No data	9 Slow

ND: no data, y: year or years, m: month or months, and y~ or m~: alive at the reported time.

Age: **under forty** (bold) and over fifty-five (underlined).

Disease course (until invasive ventilation support): ~2 years, rapid; 3–6 years, moderate; 7~ years, slow.
